# RSA prediction of high failure rate for the uncoated Interax TKA confirmed by meta-analysis

**DOI:** 10.3109/17453674.2012.672092

**Published:** 2012-04-24

**Authors:** Bart G Pijls, Marc J Nieuwenhuijse, Jan W Schoones, Saskia Middeldorp, Edward R Valstar, Rob G H H Nelissen

**Affiliations:** ^1^Department of Orthopaedics; ^2^Walaeus Library; ^3^Department of Clinical Epidemiology, Leiden University Medical Centre, Leiden,; ^4^Department of Biomechanical Engineering, Faculty of Mechanical, Maritime and Materials Engineering, TU Delft; the Netherlands; Correspondence: b.g.c.w.pijls@lumc.nl

## Abstract

**Background and purpose:**

In a previous radiostereometric (RSA) trial the uncoated, uncemented, Interax tibial components showed excessive migration within 2 years compared to HA-coated and cemented tibial components. It was predicted that this type of fixation would have a high failure rate. The purpose of this systematic review and meta-analysis was to investigate whether this RSA prediction was correct.

**Materials and methods:**

We performed a systematic review and meta-analysis to determine the revision rate for aseptic loosening of the uncoated and cemented Interax tibial components.

**Results:**

3 studies were included, involving 349 Interax total knee arthroplasties (TKAs) for the comparison of uncoated and cemented fixation. There were 30 revisions: 27 uncoated and 3 cemented components. There was a 3-times higher revision rate for the uncoated Interax components than that for cemented Interax components (OR = 3; 95% CI: 1.4–7.2).

**Interpretation:**

This meta-analysis confirms the prediction of a previous RSA trial. The uncoated Interax components showed the highest migration and turned out to have the highest revision rate for aseptic loosening. RSA appears to enable efficient detection of an inferior design as early as 2 years postoperatively in a small group of patients.

Aseptic loosening remains a major reason for revision surgery in total knee arthroplasty (TKA) (Cloke et al. 2008, SKAR report 2010). Since revision rates are generally low, it is necessary to follow up hundreds if not thousands of patients for a long period of time (10 years) to be able to detect inferior designs (Michelson et al. 1989).

A method for early detection of aseptic loosening based on few patients would be of value. Radiostereometric analysis (RSA) enables accurate measurement of migration of prosthetic components relative to bone (Selvik 1989), migration that has been shown to be associated with late aseptic loosening (Grewal et al. 1992, Karrholm et al. 1994, Ryd et al. 1995).

Although these findings are promising, few studies have actually investigated whether the RSA predictions are correct (Grewal et al. 1992, Karrholm et al. 1994, Ryd et al. 1995, Hauptfleisch et al. 2006). In TKA, the question thus remains: does TKA with increased early migration have higher revision rates for aseptic loosening?

We have already shown in a randomized RSA trial that uncoated Interax tibial components have increased early migration compared to HA-coated and cemented tibial components (Nelissen et al. 1998). We predicted that uncoated components would have a high failure rate. The aim of the present study was therefore to investigate whether this prediction of the previous RSA trial was correct. We performed a meta-analysis to evaluate the failure rate of these components.

## Material and methods

### Design of the meta-analysis, and rationale

The design was based on the Cochrane standards, and reporting of this meta-analysis follows the PRISMA guidelines (Liberati et al. 2009). In order to exclude confounding due to differences in prosthesis design, the meta-analysis was restricted to studies involving exactly the same implant as in the previously published RSA trial (Nelissen et al. 1998): the cruciate retaining (CR) Interax TKA tibial component (Howmedica/Stryker, Rutherford, NJ) with 2 polyethylene half bearings. The fixation of the components is either by cement or by bone ingrowth on uncoated or hydroxyapatite- (HA-) coated prosthetic surfaces. The cemented components had a diamond surface on the side that was within bone, whereas the uncemented components had a wire-mesh surface (2.25 mm2, corresponding to a circular pore diameter of 1,690 μm) with or without an HA coating.

**Figure 1.**
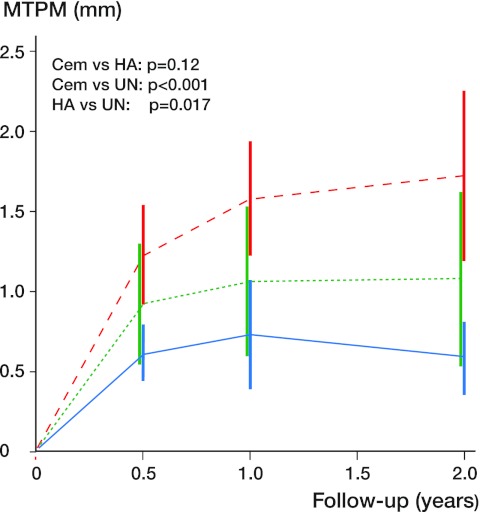
Summary of the migration results of the previous RSA trial (Nelissen et al. 1998). The plot shows the mean migration —expressed as maximal total point motion (MTPM)—with 95% CI for each type of fixation of the tibial components: red dashed line for uncoated; green dotted line for HA-coated, and blue solid line for cemented. The uncoated tibial components showed the most migration.

The outcome of interest was the number of revisions or recommended revisions for aseptic loosening of the tibial component, for each fixation separately. This outcome was compared to the early migration results of the RSA trial (Nelissen et al. 1998), which showed increased early migration of the uncoated tibial component compared to the cemented and HA-coated tibial components (Figure 1). Uncemented components show high initial migration followed by stabilization (Nilsson et al. 1991, Onsten et al. 1998, Carlsson et al. 2005, Nilsson et al. 2006, Henricson et al. 2008, Dunbar et al. 2009, Wilson et al. 2012). Thus, we also present the migration rate of MTPM (mm/year) determined from the migration measured with the postoperative RSA examination as reference (Table 1).

**Table 1.** Mean migration rate (MTPM) expressed in mm/year. The uncoated components showed the highest migration rate. The migration rate was determined from the migration measured with the postoperative RSA examination as reference

	Cemented		HA-coated		Uncoated	
Migration rate	Mean	95% CI	Mean	95% CI	Mean	95% CI
0–6 months **^a^**	1.22	0.88 to 1.57	1.84	1.07 to 2.61	2.45	1.82 to 3.10
6–12 months **^b^**	0.24	–0.34 to 0.82	0.27	–0.02 to 0.57	0.60	0.06 to 1.15
12–24 months **^b^**	–0.12	–0.31 to 0.07	0.03	–0.12 to 0.18	0.19	0.02 to 0.35

**^a ^**0–6 months: Cem vs. HA, p = 0.2; Cem vs. UN, p = 0.01; HA vs. UN, p = 0.1 (GLMM).

**^b^** 6–24 months: Cem vs. HA, p = 0.3; Cem vs. UN, p = 0.01; HA vs. UN, p = 0.1 (GLMM).

### Literature search

The literature search is the foundation on which a systematic review and meta-analysis is built. Inadequate search strategies have been shown to give biased results (Vochteloo et al. 2010). We therefore adopted a thorough search strategy in collaboration with a medical librarian, JWS. The following bibliographies were searched up to and including March 2011: PubMed, EMBASE (OVID version), Web of Science, the Cochrane Library, Current Contents Connect, CINAHL (Ebscohost-version), and Academic Search Premier (Ebscohost version). Additionally, the websites of the following medical journal publishers were searched: Elsevier ScienceDirect, Wiley Blackwell, Lippincott-Williams & Wilkins, Highwire, Informaworld/Informahealth, and Springer. To reduce the effect of any publication bias, the “gray literature” was searched up to and including March 2011: WHO International Clinical Trials Registry Platform and the proceedings of major conferences (NOF, AAOS, EFORT, ESSKA, ISTA). Furthermore, the bibliographies of included studies were hand-searched for relevant publications. Also, various lesser-known databases were searched, e.g. ScienceGov and OAIster. Finally, Google Scholar was searched.

The search involved all fields and full-text options to screen if the following component was mentioned anywhere in a manuscript (see Supplementary data for further details): “Interax” and relevant abbreviations and extensions. Since “Interax” is a registered brand name for a particular TKA model, it was assumed to be spelled out in the same way in the text of a manuscript irrespective of the language used. We did not use any language restrictions.

### Study selection

All studies were subjected to the following inclusion criteria: (1) the study comprises an original patient cohort treated with the Interax TKA (Howmedica, Rutherford, NJ); (2) the cruciate retaining Interax prosthesis with half bearings is used (posterior stabilised Interax and Interax ISA versions are excluded); (3) the type of fixation of the tibial component and the number of knees receiving this type of fixation is adequately reported; (4) the number of revisions or recommended revisions for aseptic loosening of the tibial component is reported for each fixation separately; and (5) at least 2 fixation types are compared.

2 reviewers, BGP and MJN, independently judged all studies according to these 5 inclusion criteria. In cases where the title and abstract were inconclusive, the full-text article was obtained. Any disagreement between the reviewers was resolved by re-examination and subsequent discussion to reach a consensus. Both randomized controlled trials (RCTs) and observational studies were considered for inclusion.

### Quality assessment and data extraction

The quality of each study included was independently appraised by 2 reviewers, BGP and MJN, using the Jadad scale (Jadad et al. 1996). The same reviewers independently extracted relevant data from each of the studies that were included. Any disagreement between the reviewers was resolved by re-examination and subsequent discussion to reach a consensus.

### Statistics

Before considering a meta-analysis (pooling of data), we investigated whether it was appropriate to pool the data. Studies should be similar in design and in patient population. In addition, the variability in effect size between studies should not exceed those expected from sampling error: low heterogeneity is desirable. Heterogeneity was assessed by calculating the I2 statistic, which is appropriate for a small number of studies (Higgins et al. 2002). Publication bias was assessed with a funnel plot (Sterne et al. 2000). Meta-analysis was performed with Peto odds ratio (OR) fixed-effect pooling and Mantel-Haenszel random-effects pooling for the risk difference (RD) and number needed to treat (NNT). The NNT was defined as the number of cemented tibial components that would have to be implanted in order to prevent 1 revision as compared to when uncoated components were implanted. We used RevMan software.

## Results

### Study selection and study characteristics

**Figure 2.**
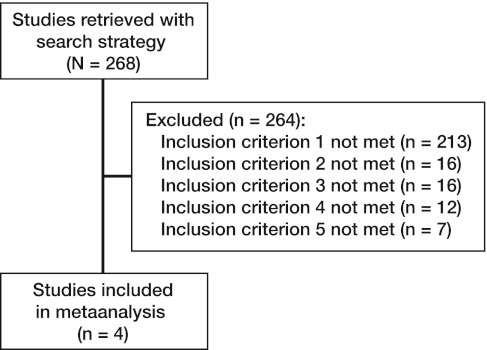
Flow diagram showing details of study selection. In cases where the title and abstract were insufficiently conclusive, the full text article was obtained.

The search strategy resulted in 268 unique hits, and 4 of these studies could be included (Gicquel et al. 2000, Stukenborg-Colsman and Wirth 2000, Wirth 2004, Petersen et al. 2005, Pijls et al. 2012) (Figure 2). 2 papers were published in English (Petersen et al. 2005, Pijls et al. 2012), 1 in German (Stukenborg-Colsman et al. 2000), and 1 in French (Gicquel et al. 2000) (Table 2). 3 studies compared the cemented component to the uncoated one (Gicquel et al. 2000, Stukenborg-Colsman and Wirth 2000, Pijls et al. 2012). 1 of these studies (Stukenborg-Colsman and Wirth 2000) was part of a thesis (Barisic and Wirth 2004), which we used for more details. 1 of these studies (Pijls 2012) was the long-term follow-up of the RSA trial (Nelissen 1998) and reported 3 revisions (2 uncoated and 1 cemented) for aseptic loosening of the tibial component. Since only 1 study with 18 TKAs (Petersen et al. 2005) compared the HA-coated tibial component to the uncoated one, no pooling was done for this comparison. The funnel plot did not show any publication bias.

**Table 2.** Characteristics of the studies included

	Cemented vs. uncoated			HA-coated vs. uncoated
Study	Pijls 2011	Gicquel 2000	Stukenborg 2000	Petersen 2005**^a^**
Type	RCT	RCT	OBS	RCT
No. of TKAs	68	96	209	18
Females, n (%)	55 (81)	NS (75)	166 (79)	15 (83)
OA, n (%)	18 (26)	NS (97)	NS (67)	18 (100)
RA, n (%)	49 (72)	NS (3)	NS (26)	0 (0)
Mean age at operation, years	66	73	68	76
Mean FU, years	7.6	2.3	6.8	2
Operation period	1993–1998	1993–1995	1991–1994	–
Deaths, n (%)	28 (42)	6 (6)	39 (19)	1 (5.5)
Lost to follow-up (%)	1 (1.5)	20 **^b^** (20)	3 (1.4)	1 (5.5)
Jadad quality score **^c^**	3	3	1	2

**^a^** Since Petersen et al. was the only study evaluating HA-coated and uncoated and included only 18 patients, no meta-analysis could be performed for the comparison of HA-coated and uncoated.

**^b^** 20 cases were lost to follow-up: 8 cemented cases and 12 uncoated cases.

**^c^** Maximum attainable score was 3 because the evaluation of revision on the radiograph cannot be blinded. RCT: randomized controlled trial; OBS: observational study; NS: not stated.

### Uncoated vs. cemented tibial component

349 TKAs were included in the meta-analysis of uncoated and cemented components. There were 30 revisions of the tibial component for aseptic loosening, of which 27 were for the uncoated components and 3 were for the cemented component.

**Figure 3.**
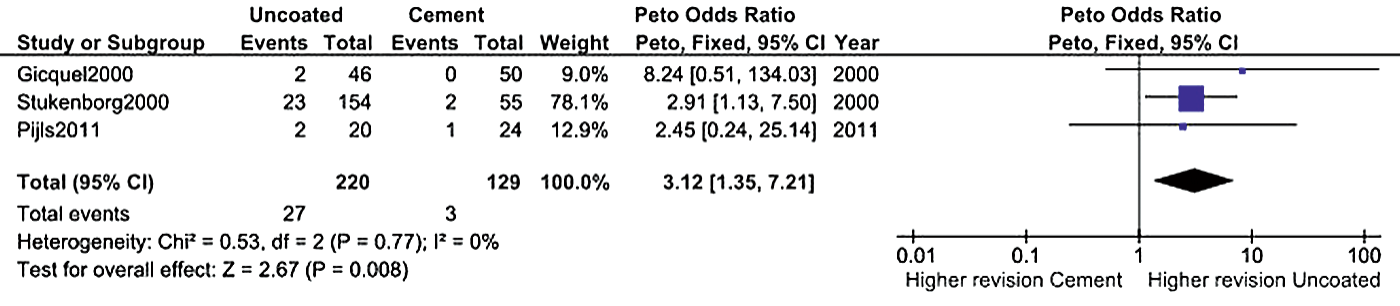
Forest plot summarising the pooled effect size of cemented and uncoated tibial components. As shown, there was a significantly (3 times) higher revision rate for the uncoated Interax tibial components than for the cemented ones.

The odds of revision due to aseptic loosening of the uncoated tibial component was 3.1 times higher than for the cemented tibial component: pooled OR = 3.1 (95% CI: 1.4–7.2) (Figure 3). The pooled risk difference was 7% (CI: 3–12) in favor of the cemented component. The number needed to treat (NNT) was 14 in favor of the cemented component (CI: 8–33). This means that for every 14 patients treated with a cemented Interax tibial component, 1 revision for aseptic loosening is prevented compared to the uncoated component.

### Risk of bias within studies

The sequence of randomization and concealment of allocation were described and appropriate in 2 studies (Gicquel et al. 2000, Pijls et al. 2012). In 1 study (Petersen et al. 2005), randomization was performed but the method and concealment was inadequately described and in another study (Stukenborg-Colsman and Wirth 2000) no randomization was performed. In the non-randomized study, the decision for implanting either a cemented or an uncoated uncemented tibial component was made by the surgeon during the operation, leading to confounding—because cemented components were used for cases with reduced bone quality (Stukenborg-Colsman and Wirth 2000). This confounding would lead to a possible underestimation of the revision rate of the uncoated uncemented tibial component. Thus, the higher revision rate for the uncoated components than for the cemented ones may have been an underestimation of the true revision rate.

In all studies blinding, was a potential source of bias. Since evaluation of radiographs is essential for the indication for a revision and the presence or absence of cement cannot be masked on the radiograph, blinding—if possible at all—was not performed in any of the studies.

The number of withdrawals and dropouts was adequately described in all studies. The number of patients who were lost to follow-up (corresponding to 8 cemented and 12 uncoated components) was high in the study by Gicquel et al. 2000 (see Table 2).

All 3 studies that compared cemented and uncoated components included all patients consecutively during the inclusion period, and thus reduced the possibility of selection bias (Stukenborg-Colsman and Wirth 2000, Gicquel et al. 2000, Pijls et al. 2012).

## Discussion

### Uncoated vs. cemented components

Our aim was to investigate whether the predictions of a previous trial using radiostereometric analysis (RSA) were correct. Since the uncoated Interax components had shown the highest migration, it was predicted that this type of fixation would have a high failure rate (Nelissen et al. 1998). The results of the meta-analysis showed a statistically significant 3 times higher revision rate for the uncoated uncemented component than for the cemented tibial component. Thus, the prediction from the previous RSA trial was correct: the uncoated tibial components showed the highest migration and had the highest revision rate for aseptic loosening. The uncoated tibicomponents also continued to migrate after 1 year, whereas the HA-coated components stabilized after 1 year. This is in accordance with a recent report by Wilson et al. (2012), which showed that tibial components can give solid fixation despite high levels of initial migration.

In the RSA trial, the high degree of migration of the uncoated uncemented tibial components was identified within 2 years in a small group of 44 patients (24 in the cemented group and 20 in the non-coated group) compared to the 349 in the meta-analysis. This emphasizes the value of RSA for the early detection of inferior TKA designs in a small series of patients (Grewal et al. 1992, Karrholm et al. 1994, Ryd et al. 1995).

It is noteworthy that none of the individual traditional clinical studies with large numbers of patients and medium-term or long-term follow-up showed a statistically significant difference in revision rates between the uncoated uncemented and cemented Interax tibial components (Gicquel et al. 2000, Stukenborg-Colsman and Wirth 2000). Only when the results of these studies were combined in a meta-analysis setting did the high revision rate in the uncoated components become clearly visible.

### Uncoated vs. HA-coated components

One of the selected studies compared the uncoated tibial component with the HA-coated component (Petersen et al. 2005). This study involved only 18 patients who were followed for 2 years. Because of the short follow-up and small patient cohort, it was not appropriate to perform a meta-analysis for the comparison of uncoated and HA-coated components. The uncoated Interax tibial component has been withdrawn from the market after the results of the RSA trial were published. Since the HA-coated component migrates less than the uncoated tibial component, a beneficial effect of the HA coating would be expected. Less migration of an HA-coated component than of a non-coated component has also been demonstrated for the Interax CR by Østgaard et al. (1999). Their migration results were similar to those of our RSA trial (Nelissen et al. 1998), despite differences in patient characteristics: all their patients were suffering from osteoarthritis, as compared to one third with osteoarthritis and two thirds with rheumatoid arthritis in our RSA trial.

### Strengths and limitations

Our search strategy was thorough and complete. This is underscored by the fact that we found 2 studies that were published in the non-English literature. Although our research question was highly specialized, i.e. fixation of a single type of TKA, we were still able to include 3 studies. This is not uncommon for orthopedic meta-analysis, even in Cochrane reviews (Jacobs et al. 2004).

The studies included were of moderate quality, mostly due to issues with blinding for the fixation method—which is a general problem in any study comparing cemented and uncemented components and was not specific to the present meta-analysis.

Publication bias generally favors the newly introduced treatment (Gotzsche 1987), the uncoated uncemented fixation in this case. Since the studies included in this meta-analysis did not find a positive effect for the uncoated components, publication bias was probably not a major factor here. Thus, we are confident that our conclusion is correct: the uncoated tibial component of the Interax has a higher revision rate for aseptic loosening.

The I-statistic was 0%, so there was no indication of statistical heterogeneity. Despite differences in patient demographics, surgical technique, or study design, all ORs were on the same side, i.e. showed higher—although not individually significantly higher—revision rates for the uncoated component, and this confirms the predictions of the RSA trial.

### Future perspectives

More than a decade ago, Liow and Murray (1997) and Muirhead-Allwood (1998) called for a more evidence-based evaluation and clinical introduction of (new) prosthetic designs and fixations. Malchau (2000) proposed a phased evidence-based introduction of new designs. Recently, a renewed call for concrete steps has been made towards such an evidence-based clinical introduction (McCulloch et al. 2009, Schemitsch et al. 2010). A disastrous design can be detected early postoperatively in a small group of patients by RSA, which therefore has the potential to play an important role in the clinical introduction of new models and fixation methods in total knee arthroplasty. For example, in vitro testing-machine studies should be followed by 2-year RSA studies in small cohorts in different institutions worldwide, followed by larger comparative studies, after which introduction to the market can be started (Malchau 2000)—with the latter also involving follow-up in national registries. In this way, a more phased prosthesis introduction to the market is guaranteed, as is currently the standard for pharmacological agents.

## Appendix

Supplementary data are available at our website (www.actaorthop.org), identification number 4935.
