# Supplementary material for: RSA prediction of high failure rate for the uncoated Interax TKA confirmed by meta-analysis
Source: Acta Orthop. 2012 Apr 24;83(2):142–7. doi: 10.3109/17453674.2012.672092 (PMC3339527; doi:10.3109/17453674.2012.672092)
Supplement: Supplementary file 1 [file ORT-1745-3674-83-142-s4935.pdf]

**Appendix: Search Strategy**

| Database | Strategy | Number of references | Number of unique references |
|---|---|---|---|
| PubMed<br>http://www.ncbi.nlm.nih.gov/entrez/query.fcgi?otool=leiden | interax | 27 | 27 |
| | | | |
| EMBASE (OVID-version)<br>http://gateway.ovid.com/ovidweb.cgi?T=JS&MODE=ovid&NEWS=N&PAGE=main&D=emez | interax.af | 37 | 21 |
| | | | |
| Web of Science<br>http://isiknowledge.com/wos | TS=interax | 25 | 7 |
| | | | |
| Cochrane Library<br>http://www3.interscience.wiley.com/cgi-bin/mrwhome/106568753/HOME | interax | 1 | 0 |
| | | | |
| CINAHL (EBSCOhost-version)<br>http://search.ebscohost.com/login.aspx?authtype=ip,uid&profile=ehost&defaultdb=cin20 | interax | 4 | 1 |
| | | | |
| Academic Search Premier (EBSCOhost-version)<br>http://search.ebscohost.com/login.aspx?authtype=ip,uid&profile=ehost&defaultdb=aph | interax | 27 | 18 |
| | | | |
| ScienceDirect<br>http://www.sciencedirect.com/ | (interax AND (knee OR prosth*)) OR TITLE-ABSTR-KEY(interax ) | 53 | 41 |
| | | | |
| Wiley-Blackwell<br>http://www3.interscience.wiley.com/cgi-bin/simplesearch | interax | 4 | 1 |
| | | | |
| Lippincott-Williams&Wilkins<br>http://ovidsp.ovid.com/ovidweb.cgi?T=JS&PAGE=main&MODE=ovidclassic&D=ovft | interax | 30 | 22 |
| | | | |
| Highwire<br>http://highwire.stanford.edu/ | interax | 33 | 24 |

| Database | Strategy | Number of references | Number of unique references |
|---|---|---|---|
| | | | |
| Informaworld/Informahealth<br>http://informahealthcare.com/ | interax | 15 | 6 |
| | | | |
| Springer<br>http://springerlink.metapress.com/app/home/search-citations.asp?wasp=3pvxje0mwm6qxm8d2hw3 | interax | 29 | 13 |
| | | | |
| Clinical Trials.gov<br>http://clinicaltrials.gov/ct2/search | interax | 0 | 0 |
| | | | |
| WHO International Clinical Trials Registry Platform<br>http://apps.who.int/trialsearch/ | interax | 0 | 0 |
| | | | |
| Google Scholar<br>http://scholar.google.com/ | interax AND knee<br>interax AND prosthesis | | |
| | | | |
| Sciencegov | interax | 1 | 1 |
| | | | |
| OAIster | interax | 2 | 2 |
| | | | |
| Scirus | interax | 75 | 73 |
| | | | |
| Proceedings | interax | 5 | 5 |
| | | | |
| **Subtotal** | | **368** | **262** |
| | | | |
| Articles citing 3 included studies using Web of Science | | 7 | 6 |
| | | | |
| **End total** | | **375** | **268** |
